# Comprehensive analysis of the polygalacturonase and pectin methylesterase genes in *Brassica rapa* shed light on their different evolutionary patterns

**DOI:** 10.1038/srep25107

**Published:** 2016-04-26

**Authors:** Weike Duan, Zhinan Huang, Xiaoming Song, Tongkun Liu, Hailong Liu, Xilin Hou, Ying Li

**Affiliations:** 1 State Key Laboratory of Crop Genetics and Germplasm Enhancement/Key Laboratory of Biology and Germplasm Enhancement of Horticultural Crops in East China, Ministry of Agriculture, Nanjing Agricultural University, Nanjing 210095, China; 2 Center of Genomics and Computational Biology, College of Life Sciences, North China University of Science and Technology, Tangshan, Hebei 063000, China

## Abstract

Pectins are fundamental polysaccharides in the plant primary cell wall. Polygalacturonases (PGs) and pectin methylesterases (PMEs), major components of the pectin remodeling and disassembly network, are involved in cell separation processes during many stages of plant development. A comprehensive study of these genes in plants could shed light on the evolution patterns of their structural development. In this study, we conducted whole-genome annotation, molecular evolution and gene expression analyses of PGs and PMEs in *Brassica rapa* and 8 other plant species. A total of 100 PGs and 110 PMEs were identified in *B. rapa*; they primarily diverged from 12–18 MYA and PMEs were retained more than PGs. Along with another 305 PGs and 348 PMEs in the 8 species, two different expansion or evolution types were discovered: a new branch of class A PGs appeared after the split of gymnosperms and angiosperms, which led to the rapid expansion of PGs; the pro domain was obtained or lost in the proPMEs through comprehensive analyses among PME genes. In addition, the PGs and PMEs exhibit diverged expression patterns. These findings will lead to novel insight regarding functional divergence and conservation in the gene families and provide more support for molecular evolution analyses.

The plant cell wall is a complex and dynamic structure composed of diverse polysaccharides and structural proteins. Polysaccharides represent up to 95% of the plant cell wall mass, whereas proteins account for only 5–10%^1^. The polysaccharides of plant cell walls are divided into three types: cellulose, hemicellulose, and pectin^2^. Pectins, a major component of plants’ primary cell walls for maintaining cellular structural integrity, are characterized primarily by the linear backbone of the α-1, 4-linked galacturonic acid residues^3^,^4^, and they appeared after the divergence of chlorophyta and charophyte^5^,^6^.

The pectin network is systematically disassembled at many stages during plant development, such as organ abscission, fruit ripening, and pod and anther dehiscence^7^,^8^. The dismantling and modification of the pectin network is performed by a wide range of hydrolytic enzymes. These enzymes are classified by the substrate specificity or mode of action into various classes, including polygalacturonase (PG; EC 3.2.1.15), pectin methylesterase (PME; EC 3.1.1.11) and pectin lyase (EC 4.2.2.2)^9^. PMEs play a central role in both pectin remodeling and disassembly and in the firming and softening of the cell wall^10^,^11^. PGs, one of the largest hydrolase families, are important for pectin disassembly^7^,^12^. The action of PMEs is required prior to the action of PGs^13^. Previous studies have examined PG and PME functions during plant development processes, particularly during pollen development and pollen tube growth: *Arabidopsis* PMEs, which function in pollen tube growth, were inactivated by VANGUARD1 (VGD1), resulting in unstable and poorly growing pollen tubes^14^, and for PGs, the knockout of three *Arabidopsis* PGs led to the failure of pollen grain separation as well as silique and anther dehiscence^8^. *BcMF2, BcMF6* and *BcMF9*, three *Brassica campestris* PGs, have been shown to be critical for pollen wall development^15^. Galacturonic acid, one of the major products of pectin disassembled by PGs and PMEs, can increase ascorbate accumulation in fruits^16^.

To date, genomic analyses have shown that PGs and PMEs belong to large gene families. In *Arabidopsis*, a total of 66 genes have been suggested to potentially encode PGs, and the same number of genes have been suggested to encode PMEs^17^,^18^. PG genes in plants are divided into three distinct groups (classes A, B and C)^17^, whereas PME genes are classified into two types: the type I PME (referred as proPME), which contains an additional pro domain besides the PME domain, and the type II PME (referred to as PME), which contains only a PME domain^11^,^19^. The pro domain shares similarities with the PMEI domain, suggesting that there is a target PME on the cell wall to correct the folding of the enzyme and inhibit PME activity^20^. The cleavage of the pro domain occurs before mature PME functioning^20^. Furthermore, the pro domain functions in mediating the release of mature PME from the Golgi^21^. As we know, higher plant lineages have undergone polyploidization throughout their long evolutionary history^22^. These two large families experienced expansion and functional divergence through whole-genome duplication (WGD) in angiosperm genomes. Does gene function contribute to the frequency of gene loss during fractionation after WGD? The gene dosage hypothesis predicts that genes that are in networks or that function in a dose-sensitive manner should be retained^23^. Notably, the rapid expansion of the PG gene family in angiosperms is largely due to the gene duplication of class A PG genes^24^. However, the divergence of class A PGs and the evolution of the two types of PMEs in plants remain unclear.

*Brassica rapa*, a diploid species, shares *Arabidopsis’* complex history (γ, β and α events) and experiences an additional whole-genome triplication (WGT) event 13–17 million years ago (MYA)^25^,^26^. Specifically, the *B. rapa* genome has undergone considerable fractionation (i.e., duplicate gene loss) because of WGT, which provides an opportunity to study the molecular evolution of PG and PME genes. In addition, the recent availability of genome sequences for many plant species enables comparative genome analyses to make inferences regarding their origin and evolution. Therefore, we performed a comparative genome analysis of PG and PME genes across 8 other representative plant species, including algae (*Chlamydomonas reinhardtii*), bryophytes (*Physcomitrella patens*), lycophytes (*Selaginella moellendorffii*) and angiosperm plants (*Amborella trichopoda, Vitis vinifera, Populus trichocarpa, Carica papaya* and *A. thaliana*). Algae are the ancestor of land plants, containing both protistan and higher taxa^27^. Bryophytes are the closest extant relatives of early land plants^28^. Lycophytes are early vascular plants with a dominant sporophyte generation^29^, and the angiosperm plants possess more complex organ systems and structures.

In this study, we conducted a genome-wide analysis of PG and PME genes in the *B. rapa* genome and a comparative genomic analysis of these genes with 8 other plant species. Two different expansion types were found in the evolutionary histories of PG and PME genes. Overall, the findings of this study are as follows. (i) *B. rapa* PGs and PMEs primarily diverged from their *A. thaliana* orthologs 12–18 MYA. During this event, PMEs were retained more than PGs. (ii) The chromosome distribution, gene sequences, gene structures, and phylogenetic relationships of PGs and PMEs were identified in *B. rapa*. (iii) The evolution types of PGs and PMEs were explored: the appearance of a new branch of class A PGs after the split of gymnosperms and angiosperms brought about the rapid expansion of class A PGs, and the pro domain may have been obtained or lost in the proPMEs. (iv) Finally, the expression patterns of both PGs and PMEs were divergent, and obvious tissue-specific expressions were detected in these genes in *A. thaliana* and *B. rapa*.

## Results

### Copy number variation of PG and PME genes following WGT in *B.* r*apa*

The *B. rapa* genome has undergone WGT since the divergence from the last common ancestor with *A. thaliana*, establishing that the three sub-genomes can be distinguished by the degree of fractionation (gene loss)^25^,^30^. To investigate the copy number variation of PGs and PMEs genes in *A. thaliana* and *B. rapa* during the *Brassica*-specific WGT event, the HMM software package was used to identify the putative PG and PME proteins in *A. thaliana* and *B. rapa,* respectively. The candidates were then subjected to the Pfam, SMART and NCBI databases to confirm the presence of the PG and PME domains. Sixty-seven PGs and 67 PMEs (including 44 proPMEs) were identified in *A. thaliana.* However, we removed one gene (AT1G23470.1) that is defined as a pseudogene and added two genes (AT4G32375.1 and AT5G49215.1) as PGs, on the basis of previous reports^17^. In addition, we changed a PME gene to a proPME gene (AT3G27980.1) and added a PME gene (AT3G10720.1), compared on the basis of previous reports (Tables S1 and S2)^11^. Simultaneously, 100 PGs and 110 PMEs (including 68 proPMEs) were identified in *B. rapa* after predicting protein coding open reading frames with parameters optimized for *A. thaliana* by the FGENESH program (Tables S3 and S4).

**Figure 1:**
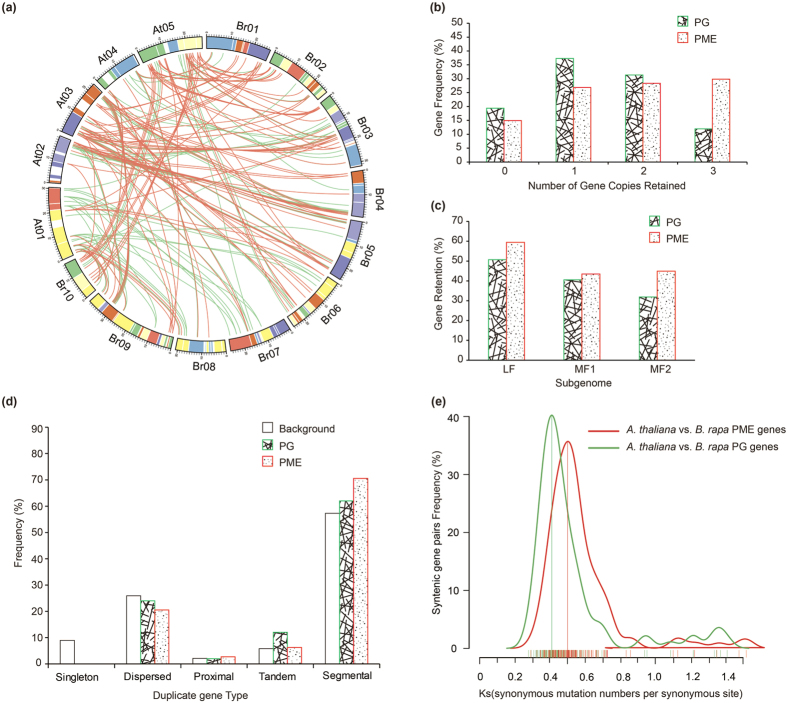
Retention of pectin methylesterase (PME) and polygalacturonase (PG) homologous copies in the syntenic region of *Brassica rapa* and *Arabidopsis thaliana* and their *K*_*s*_ values. (**a**) Collinear correlations of PG and PME genes in the *A. thaliana* and *B. rapa* genomes by Circos. The *B. rapa* and *A. thaliana* chromosomes are colored according to the inferred ancestral chromosomes, following an established convention. (**b**) Copy numbers of PG and PME genes after genome triplication and fractionation in *Brassica rapa*. (**c**) Retention of homolog copies of PG and PME genes in the three subgenomes (LF, MF1 and MF2) in *B. rapa*. LF: least fractionized; MF1: moderately fractionized; MF2: most fractionized. (**d**) The different duplicated types (singleton, dispersed, proximal, tandem and segmental) were counted in *B. rapa*. Open boxes indicate the whole genome level, filled lines boxes indicate PGs, and filled points boxes indicate PMEs. (**e**) The distribution of Ks values for PG and PME genes in *A. thaliana* and *B. rapa*. The lines representing PG and PME genes are red and green, respectively.

In this study, 196 and 199 *B. rapa* syntenic regions for the *A. thaliana* PG and PME genes, respectively, were identified using syntenic analysis with MCScanX (Figure S1). In detail, five and two *A. thaliana* PG and PME genes, respectively, were found in regions present in *B. rapa* in two syntenic blocks; the remainder were in three syntenic blocks. Wang *et al*. reported that each of the syntenic blocks can be distinguished as one of the three sub-genomes LF, MF1, or MF2 in BRAD (the *Brassica* database)^25^. Ninety-nine PG and 107 PME gene homologs were identified in these sub-genomes (Table S5). Among these copies, 89 (90%) PG homologs and 102 (95%) PME homologs were located in the syntenic regions, and the remaining genes were identified at non-syntenic sites (Fig. 1a and Figure S1). Overall, 51% (102/199) of the PME genes have been retained in the syntenic regions compared with 45% (89/196) of the PG genes (Figure S1). Then, we specifically compared the retention of PG genes relative to the retention of PME genes by counting the number of gene copies and the different distributions of the three sub-genomes. Most PME (58%) genes were retained in two or three copies, which is significantly greater than the retention of any of the PG (43%) genes, whereas fewer genes were completely lost for PME (14.9%) than for PG genes (19.4%; Fig. 1b).

The proportion of PG and PME homologs retained varied among the three sub-genomes (Fig. 1c). In the LF sub-genome, more PG and PME homologs were retained than in the other two sub-genomes. This finding is consistent with a previous report by Wang *et al*.^25^, who found that the degree of retained genes in these three sub-genomes (LF, MF1 and MF2) was sequentially reduced, whereas the PME homologs retained more in the MF2 sub-genome than in the MF1 sub-genome (Fig. 1c). The distribution of retained genes among the three sub-genomes is higher for PME gene homologs than for PG gene homologs, which once again confirmed PMEs were more preferentially retained than PGs during diploidization following WGT in *B. rapa*.

### Duplication and *Ks* analysis of PG and PME genes in *A. thaliana* and *B. rapa*

*A. thaliana* contained 24 conserved ancestral genomic blocks (GBs, labeled A-X)^31^. Due to fractionation, 71 GBs were identified in *B. rapa*^26^. To further understand the duplication types of PG and PME genes, all of them were identified non-randomly on the GBs of ten *B. rapa* chromosomes (Figure S2a). On the one hand, the PG and PME syntenic orthologs between *A. thaliana* and *B. rapa* were identified on the same GBs, indicating that segmental duplication events had occurred in these genes (Fig. 1a). On the other hand, a total of 8 PG gene pairs and 12 PME gene pairs were found to have experienced a tandem duplication event at the physical location in the individual chromosome with no more than one intervening gene (Figure S2a). Among them, a number of tandem duplicated genes were found: more than 3 in 4 PG cluster regions and 3 PME cluster regions (Figure S2b,c). Furthermore, duplicated types for these genes were identified by the MCScanx program. We noted that more tandem and segmental duplication was contained in the PGs and PMEs than in the whole genome level in *B. rapa* (Fig. 1d).

To estimate the divergence years of *B. rapa* PGs and PMEs relative to their respective orthologs in *A. thaliana*, their *K*_*s*_ (synonymous substitution rates) and *K*_*a*_ (nonsynonymous substitution rates) values were calculated. In total, 89 PG and 101 PME syntenic gene pairs were analyzed (Table S6). The *K*_*a*_/*K*_*s*_ ratios were less than 1, indicating that purifying selection occurred in the PG and PME genes. The *K*_*s*_ values of the *B. rapa* PG genes relative to the *A. thaliana* orthologs ranged from 0.2 to 0.6 and focused on 0.4 (~13.3 million years), whereas those of the PME orthologs ranged from 0.3 to 0.7 and focused on 0.5 (~16.7 million years; Fig. 1e). Based on the *K*_*s*_ values for all syntenic orthologs in *B. rapa* relative to *A. thaliana* (~0.42–0.45, ~14.5 million years)^26^, we conclude that the divergence of PG and PME genes was concurrent with the *Brassica*-specific WGT event (13–17 MYA)^25^. Specifically, the *BraPMEs* experienced a duplication event three million years earlier than *BraPGs* (Fig. 1e).

### Expansion and structural characteristics of PG and PME genes in *B. rapa*

To investigate the extent of the lineage-specific expansion of the PG genes in *B. rapa*, the phylogenetic relationships among the 100 *B. rapa* PGs were reconstructed. The phylogenetic tree showed that the PGs formed three distinct clades (class A: red; class B: blue; and class C: yellow) with 100% bootstrap support (Figure S3a). In *B. rapa*, the class A, B and C PG genes contained 79 members, 20 members and 1 member, respectively, whereas in *A. thaliana*, class A, B and C PG genes contained 53 members, 13 members, and 1 member, respectively. Next, the synteny of PG genes between *A. thaliana* and three sub-genomes in *B. rapa* was analyzed. There were 42 ancestral class A and 12 class B PG genes on the conserved collinear block (Table S5). After the split, *B. rapa* gained 7 and 3 genes and lost 11 and 1 genes in classes A and B, respectively, resulting in the different expansion of these PGs. Because of the *Brassica*-specific WGT event, the gene numbers of these two classes in *B. rapa* was greater than that in *A. thaliana*. Notably, each *B. rapa* and *A. thaliana* genome had one class C PG gene and neither a gain nor a loss of genes, but the two genes were not on the conserved collinear block (Table S5).

To discover the motifs shared among the three classes of BraPG proteins, we identified 10 motifs using the MEME program^32^. In the phylogenetic trees, the BraPG proteins that clustered into the same subclass generally shared similar motif compositions. However, the N- and C-terminal parts of these proteins were divergent among the three classes (Figure S3b). The gene structure was also analyzed. The red box in Figure S3c represents the GH28 domain, which took possession of most exons. Highly variable gene structures were observed in half (40 of 79 class A PGs had four or fewer exons) of the *B. rapa* class A PGs, whereas conservative gene structures were found among most (16 of 20 class B PGs had five or more exons) of the class B PGs (Figure S3c).

**Figure 2:**
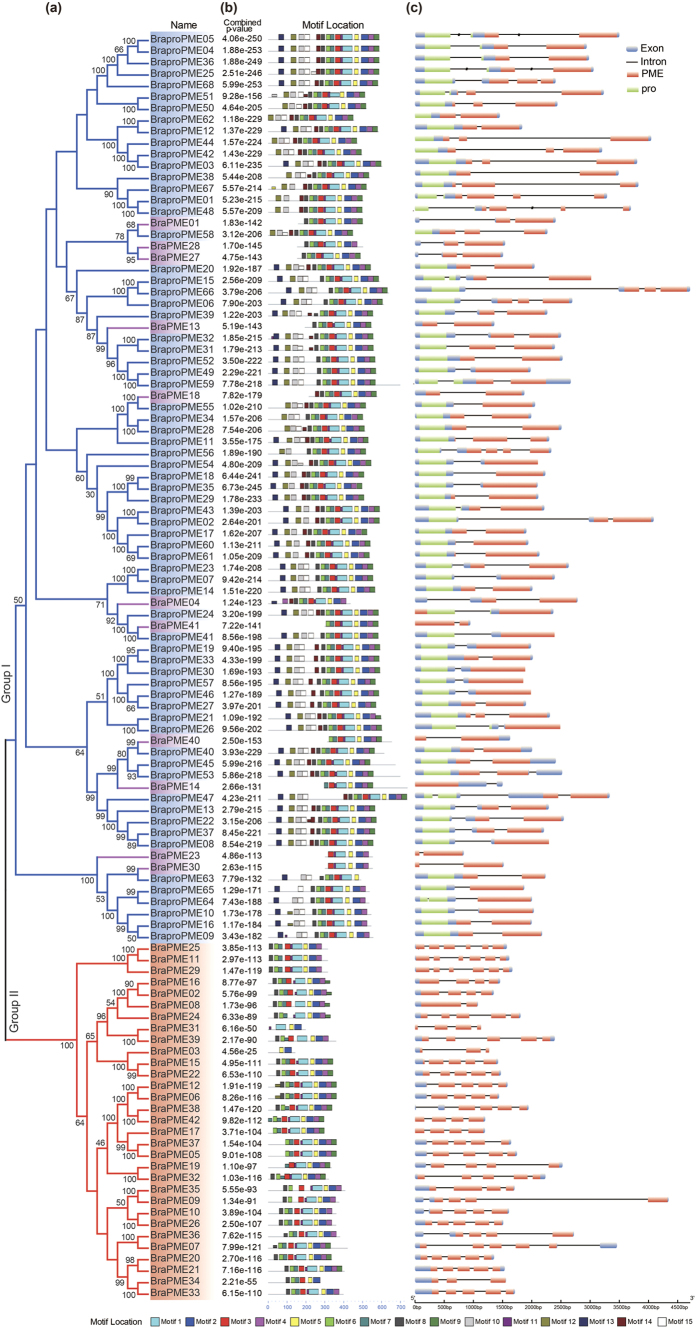
An analytical view of the pectin methylesterase (PME) gene family in *Brassica rapa*. The following parts are shown from left to right. (**a**) Protein maximum-likelihood (ML) tree: this tree was constructed using the ML method, and bootstrap values were calculated with 1000 replications using MEGA 5.2; type I PMEs are colored blue, type II A PMEs are colored red and type II B PMEs are colored purple. (**b**) Protein structure: the search for the common motifs shared among the PME proteins of each group was performed using MEME; the clade situation was in the bottom. (**c**) Gene structure: the pro and PME domains are highlighted by green and red boxes; introns are shown as lines.

A phylogenetic analysis was also conducted to study the evolutionary relationship between the type I and type II PME genes. Two groups of PMEs in *B. rapa* were evident based on the bootstrap values: all type I and 11 of the type II BraPMEs were clustered into group I, and the other type II BraPMEs belonged to group II (Fig. 2a). Then, phylogenetic relationships among all the type II BraPMEs were reconstructed. The type II BraPMEs formed two distinct clades, defined as A (red line, Figure S4a) and B (purple line, Figure S4a) with 100% bootstrap support. The same cluster was also found in *A. thaliana* (Figure S4b,c). To further investigate the evolutionary patterns of these PME genes, synteny analyses among *A. thaliana* and three sub-genomes in *B. rapa* were performed. Three conserved collinear blocks flanking two type I orthologous AtPMEs were identified among three *B. rapa* sub-genomes (Figure S4d). Then, these three regions were investigated using CoGE (short for comparative genomics; Table S7, Figure S4e). Most (9/11) type II B *BraPME* genes, such as *BraPME04,* were orthologous to type I *AthPME* genes (Figure S4f and Figure S5a–e). However, the *BraPME28* special type II B gene was retained from an ancient gene with the type II B *AtPME* gene (Figure S5f). Among all of the *BraPME*s, we also found that three type I *BraPME* genes were orthologous to the type II B *AtPME* gene (Figure S5g). Meanwhile, all 31 type II A *BraPME* genes and 19 type II A *AtPME* genes were retained as the orthologous genes after the split, suggesting that the type II A PME genes expanded conservatively in these two plants (Table S5).

**Figure 3:**
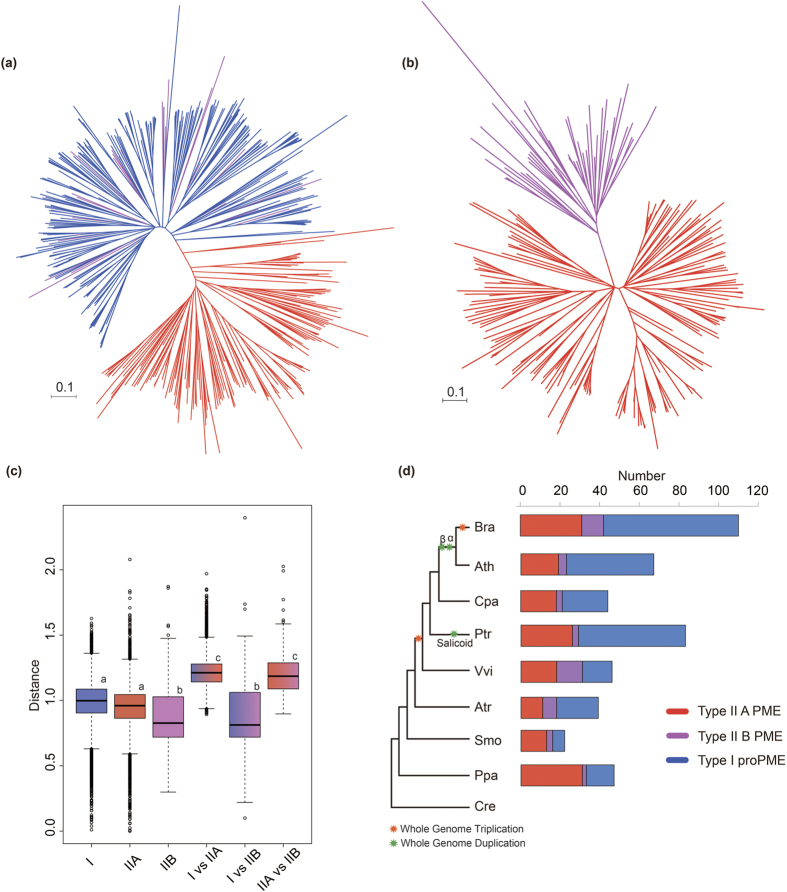
Phylogenetic relationships among 458 pectin methylesterase (PME) genes (**a**) and 213 type II PME genes (**b**); nucleotide distance among different types of PMEs (**c**); comparison of the copy numbers of PME genes in representative species (**d**). Types I, II A and II B PMEs are shaded blue, red and purple, respectively. In (**c**), the boxplot shows the median (black line), interquartile range (box), and maximum and minimum scores (whiskers) of each dataset. Different letters indicate statistical significance (P < 0.05) as determined by Duncan’s Test. In (**d**), the abbreviated species names are *C. reinhardtii* (Cre), *P. patens* (Ppa), *S. moellendorffii* (Smo), *A. trichopoda* (Atr), *V. vinifera* (Vvi), *P. trichocarpa* (Ptr), *C. papaya* (Cpa), *B. rapa* (Bra) and *A. thaliana* (Ath). The α, β, γ, and salicoid duplications and the *Brassica*-specific triplication are indicated on the branches of the trees, according to the Plant Genome Duplication Database.

In addition, the structural characteristics of BraPMEs were analyzed. All 15 conserved motifs were detected in BraPMEs. Almost all BraPMEs had motifs 1–6, which corresponded to highly conserved PME domains, whereas motifs 10, 12, 13 and 15 were found in type I BraPMEs, corresponding to pro domains (Fig. 3b). For the BraPMEs, variable gene structures were observed. In detail, among 32 type II A BraPMEs, 22 had five or more exons, and 6 had four exons. Among 68 type I *BraPMEs* and 11 type II B *BraPMEs*, 34 type I genes and 7 type II B genes had two exons, and 25 type I genes and 4 type II B genes had three exons (Fig. 3c). Thus, the type II B PME genes had a gene structure more similar to the type I genes than the type II A genes. In summary, we concluded that the type II B PMEs had a closer relationship with the type I PMEs.

### Evolution pattern of PME genes in plants

To investigate the evolution pattern of the PME gene family in the plant kingdom, we collected 7 other plant species, including 3 eudicots (*C. papaya, P. trichocarpa* and *V. vinifera*), 1 basal angiosperm (*A. trichopoda*), 1 lycophyte (*S. moellendorffii*), 1 physcomitrella (*P. patens*) and 1 chlorophyta (*C. reinhardtii*), for a comparative analysis. In this study, a total of 281 PMEs were identified in all of these species by the Pfam program (Table S8), but no PMEs were detected in *C. reinhardtii*. Therefore, the PME gene family may be unique to land plants. To classify these PMEs, phylogenetic trees were constructed for each species (Figure S6). The phylogenetic tree showed that all PMEs were divided into two distinct groups (group I: type I and type II B PMEs; group II: type II A PMEs) with high bootstrap value support, which is consistent with the result for *B. rapa* and *A. thaliana* (Figure S6). Using the ML method, we reconstructed the phylogenetic trees of the 458 PMEs and 213 type II PMEs, respectively. In both trees, the type II B PMEs were separated from the type II A PMEs (Fig. 3a,b). Furthermore, we calculated the nucleotide distance among these three types. The nucleotide distance of type II B genes was the smallest, followed by type II A and type I. Notably, the nucleotide distance between the type II B and type I PMEs was smaller than that between the type II B and type II A PMEs, whereas the nucleotide distance between type II A and type I was similar to that between type II A and type II B (Fig. 3c). All of these results indicated that type II B PMEs had a closer relationship with type I than with type II A PMEs.

**Figure 4:**
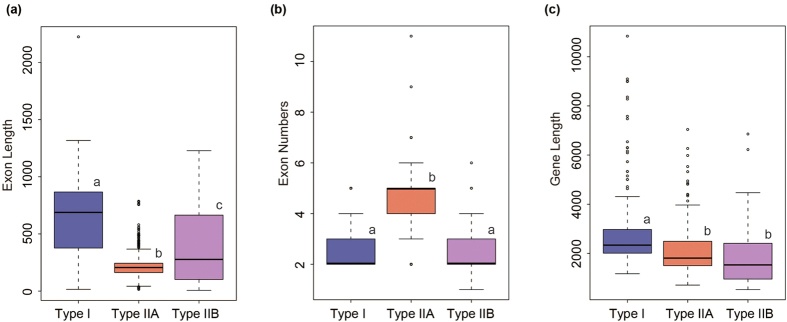
Boxplot of the exon length (**a**), exon numbers (**b**) and gene length (**c**) of the pectin methylesterase (PME) genes in representative species. Type I, II A and II B PMEs are highlighted by blue, red and purple boxes, respectively. Different letters indicate statistical significance (P < 0.05) as determined by Duncan’s Test.

Gene exon-intron structure analyses of the PMEs were conducted in 8 plant species (Table S9). The average exon length of type I PMEs was larger than that of type II PMEs, whereas the interquartile range of type II B PMEs was similar to that of type I PMEs (Fig. 4a). Obviously, type II A PMEs possessed more than 4 exons with conservative gene structures, whereas both type I and type II B PMEs possessed 2–3 exons (Fig. 4b). In addition, the average gene length of proPMEs (type I) was larger than that of PMEs (type II A and B) due to the loss of the pro domain (Fig. 4c). Using a graphical display, we depict the PME gene structures of *B. rapa* (Fig. 2c), which further show that type II B PMEs had a close relationship with type I PMEs.

The markedly different types in the PME gene family among bryophytes, lycophytes and angiosperms indicated that type II B was found from the bryophytes (Fig. 3d). Furthermore, the gene numbers of type II B PMEs were varied in different plant species. *V. vinifera* (28%) and *B. rapa* (11%) have more type II B PMEs than ‘the other plant species analyzed, indicating that the rate of the pro domain loss in proPME genes was associated with different WGD events. Compared with the type II PMEs, the type I PMEs expanded more rapidly in these species (Fig. 3d and Table S10). To further understand the effects of WGD on the expansion and evolution of gene families in plant genomes, the PME orthologs among all 8 species were analyzed using the OrthoMCL program^33^. All 1311 orthologous gene pairs and 910 co-orthologous gene pairs of PMEs were identified. There were more orthologous gene pairs in type I than type II PMEs among these species (Table S11). To further obtain insight into the correlation of these genes, the networks of all orthologous gene pairs were constructed using the Cytoscape program (Figure S7). These PME orthologous gene pairs were also clearly divided into two groups: a type II A PME group and a type II B and type I PME group (Figure S7a,b). This finding suggested that type II B PMEs might have originated from type I PMEs and that the pro domain may have been lost through a selective pressure on type I PMEs.

**Figure 5:**
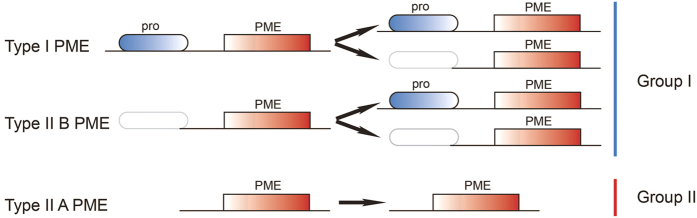
The evolutionary pattern of pectin methylesterase (PME) genes in the plant kingdom. The rounded blue box indicates the pro domain, the red box indicates the PME domain, and the non-colored rounded box indicates the lost pro domain.

In summary, we inferred a possible evolutionary model of the PME gene family (Fig. 5). i) The PMEs were clustered into two groups dating back to the divergence of *P. patens*. Group I contained all proPMEs and part of the PMEs without the pro domain, whereas group II contained PMEs only with PME domain. ii) Group I genes may have obtained or lost the pro domain during evolution.

### Evolutionary history and structural characteristics of the PG gene family in plants

**Figure 6:**
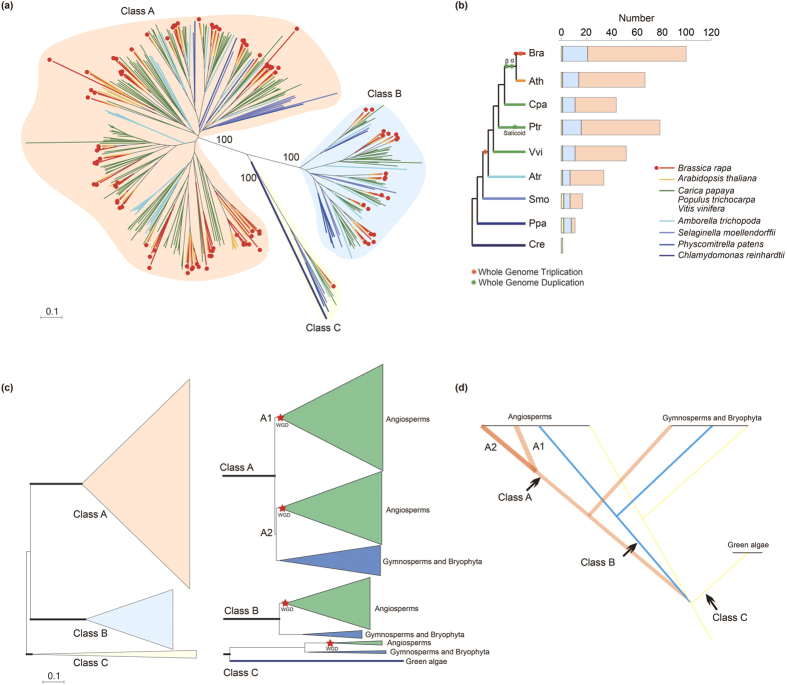
The duplication origin of polygalacturonase (PG) genes in plants. (**a**) Phylogenetic tree of the 405 PGs from nine selected plant species. The tree was constructed using the neighbor-joining method. Numbers at the internal branches leading to the three PG classes indicate the bootstrap support from 1000 replicates. (**b**) Comparison of the copy numbers of PGs in these plant species. Class A, B and C PGs are shaded red, blue and yellow, respectively, and different species have different colors. (**c**) Tree summarizing our understanding of the phylogenetic relationships of PGs. The red stars denote the inferred angiosperm whole-genome duplication. (**d**) Schematic representation of the duplication history of PGs in plants. The orange, blue and red lines indicate class A, B and C PGs, respectively.

To further clarify the evolutionary history of the PG gene family in plants, PG genes were collected from the other 7 plant species studied in this work for the comparative analysis. A total of 238 PG genes were identified, and only one gene was found in *C. reinhardtii* (Table S12). Then, the phylogenetic relationships among all PGs were reconstructed. All PGs in these 9 plant species were divided into three distinct classes, as the *B. rapa* (classes A, B and C), with 100% bootstrap support (Fig. 6a). To analyze the relationships among these three classes, the nucleotide distance was used. The boxplot shows that the nucleotide distance of class B and C PGs was smaller than that of class A PGs (Figure S8), indicating that the degree of sequence divergence among class A PGs was higher than that among class B and C PGs. The nucleotide distance between class A and class C was the largest, and a similar nucleotide distance between PG classes was found in classes A and B and classes B and C (Figure S8). This finding indicated that class B had a close phylogenetic relationship with class A and class C, consistent with the result of the phylogenetic trees.

The gene exon-intron structures of the PGs were also analyzed in these species (Table S9). The average exon length of class C was greater than that of the class A and class B PGs (Figure S9a). The fewest exons were found in class C, and the most exons were found in class B (Figure S9b). Notably, the scope of the exon numbers and the average gene length of class A PGs were wider and shorter than those of the other classes (Figure S9b,c). The gymnosperms and bryophyte species (*S. moellendorffii* and *P. patens*) contained more exons (~7.6) than all angiosperm species (~5.83; Table S9). In addition, highly variable gene structures were observed in a branch of class A PGs (Figure S9b). This part of the PG genes had fewer exons and a shorter gene length than the other class A PGs, indicating that the part of the class A PG genes may be differentiated from angiosperms.

The different degrees of gene numbers of the three classes of the PG gene family were marked among the 9 plant species (Fig. 6b and Table S10). One class C PG gene was found in *C. reinhardtii*, suggesting that PG gene expansion occurred after the divergence of green algae. Highly conserved copy numbers of the class C PGs were found in these species. All species had only one class C PG gene, except *S. moellendorffii* and *P. patens,* both of which had two PGs. Similar to class C PGs, the conserved copy numbers of the class B PGs in the 8 species were observed (no class B PGs were identified in *C. reinthardtii.*). However, the copy number of class A PGs showed variations, particularly after the divergence of the gymnosperms and angiosperms. In combination with the phylogenetic relationships, we found that class A could be divided into two major groups, A1 and A2. A2 contained PGs from all species except green algae, whereas A1 contained PGs only from angiosperms (Fig. 6c), showing that A1 PGs might have diverged from angiosperm class A2 PGs, and the rapid expansion of class A PG genes may have primarily caused PG genes to expand in angiosperm plants.

The ortholog groups of all PGs among these 9 plant species were also analyzed. All PG orthologs could be divided into 26 groups (Table S13), whereas only one group was found in all 9 species, which was the class C PGs (Figure S10). Eighteen specific groups were found only in the selected angiosperms, and 9 were found only in core eudicots. Furthermore, the class A1 PG genes (11 groups) were included only in the 18 angiosperms groups, and 8 class A1 PGs groups belonged to the core eudicots (Figure S10 and Table S13). We concluded that the differentiation of class A PGs occurred in angiosperms, particularly after WGD. The evolutionary history of PGs in the plant kingdom was then constructed in accordance with our findings: class B might have originated from class C, which was the initial group; then, class A separated from class B in angiosperm plants (Fig. 6d).

### Divergence of selective pressure on PG and PME genes

In a previous report, class A PG genes were under more relaxed selection constraints than the class B PG genes in *A. thaliana* and *P. trichocarpa*^24^. To infer the influence of selection on the expansion of class A PGs in *B. rapa*, we determined the change in the selective pressure between the two classes using ML codon models in PAML software. The log-likelihood values under the one-ratio and two-ratio models for *B. rapa* PGs were lnL = −96568.388342 and −96509.301975, respectively (Table S14). The LRT showed that the two-ratio model was not equal to the null model (one-ratio model), suggesting that the selective pressure differed significantly between the two classes (P < 0.0001). Under the two-ratio model, the ω values for *B. rapa* classes A and B were 0.2751 and 0.1664, respectively, indicating that the *B. rapa* class A PGs were under more relaxed selection constraints than the class B PGs. This finding is consistent with that for *P. trichocarpa*^24^. These results are consistent in that the relaxed pressure may have led to class A PGs having more diverse functions.

In the same way, different selective pressures between the two groups of PMEs in plant species, including *B. rapa, A. thaliana* and 6 others, were also identified (Table S14). For *B. rapa* PMEs, the LRT showed again that the two-ratio model was not equal to the null model (one-ratio model), suggesting that the selective pressure differed significantly between the two groups (P < 0.0001). Under the two-ratio model, the results suggested that the *B. rapa* group II PMEs were under more relaxed selection constraints than the group I PMEs. Similar to *B. rapa*, the group II PMEs in eudicots also had higher ω values than that of their group I PMEs. However, *A. trichopoda* group I PMEs are under more relaxed selection constraints than group II PMEs, and *S. moellendorffii* group I PMEs showed a similar selective pressure to group II PMEs. We speculated that group I PMEs underwent functional divergence in *A. trichopoda*, whereas in eudicots, the group II PMEs gained more diverse functions, and group I (type II B and type I) was relatively stable.

### Comparative expression pattern analysis of PG and PME genes between *A. thaliana* and *B. rapa*

To detect the functional divergence of PGs and PMEs, the expression of all genes was compared in different tissues between *A. thaliana* and *B. rapa*, including roots, stems, leaves, flowers and siliques; callus was studied only in *B. rapa,* and mature pollen was studied only in *A. thaliana* (Tables S15 and S16). A total of 51 (76%) *AtPGs* showed high expression (mean-normalized value >1) in at least one of the six tissues (Figure S11a), whereas more *AtPME* genes (58; 87%) were highly expressed (Figure S11b). Among these genes, more genes (38 *AtPGs* and 35 *AtPMEs*) were highly expressed in mature pollen than other tissues, and 5 *AtPGs* and 9 *AtPMEs* were highly expressed only in mature pollen, indicating that these genes were important during pollen development. Tissue-specific expression profiles were also analyzed for *BraPGs* and *BraPMEs*: 20 *BraPGs* and 32 *BraPMEs* were highly expressed (FPKM value >10) in flowers, and most *BraPGs* and *BraPMEs* had no or low expression in other tissues (Figure S12a,b).

**Figure 7:**
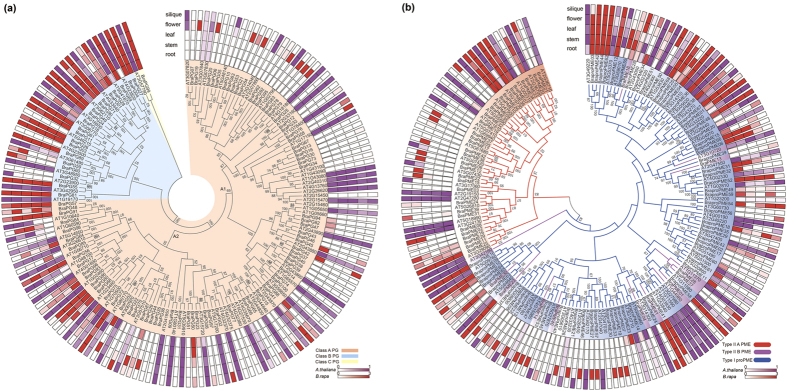
Expression pattern analysis of polygalacturonase (PG) and pectin methylesterase (PME) genes in *Arabidopsis thaliana* and *Brassica rapa*. Phylogenetic relationships and expression patterns in PGs (**a**) and PMEs (**b**). Class A, B and C PGs are highlighted red, blue and yellow, respectively, whereas type I, II A and II B PMEs are highlighted blue, red and purple, respectively. The gene expression was determined by the AtGenExpress Visualization Tool and *B. rapa* RNA-Seq data. The bar at the bottom of each heat map represents the relative expression values.

Then, we screened the expression pattern in five tissues of genes on the phylogenetic tree of all PGs and PMEs to investigate whether the functions of the homologous genes were divergent (Fig. 7a,b). For PG genes, all class C and most class B and A2 genes had high expression levels, suggesting significant roles of these genes in plant development. Most class A1 *BraPGs* exhibited little or no expression in any tissue, and 11 A1 *BraPGs* were expressed only in the flower. However, part of the A1 *AtPGs* functioned in all tissues, indicating that *BraPGs* may have obtained specific functions in flower development and lost some functions after the duplication events (Fig. 7a). For PME genes in both group I and group II, the functions of part of the homologs were similar, but others were divergent. In addition, the expression patterns in the group II PMEs were more divergent among the within-group than the group I PMEs. This result may be due to the group II PMEs being under more relaxed selection. Compared with homologous type I proPMEs, most type II B PMEs were expressed divergently (Fig. 7b), showing that the pro domain might have an effect on the expression of proPMEs.

## Discussion

Polygalacturonases and pectin methylesterase are involved, directly or indirectly, in diverse physiological processes associated with both vegetative and reproductive plant development^7^,^20^. PMEs play a central role in both pectin remodeling and pectin disassembly^11^, whereas PGs are important for pectin disassembly^7^.

The gene balance hypothesis predicts that genes whose products participate in macromolecular complexes or in transcriptional or signaling networks were more likely to be retained, thus avoiding the network instability caused by the loss of one member^23^,^34^. In this study, 100 PGs and 110 PMEs were identified in the *B. rapa* genome, and they contained more duplications than the *B. rapa* whole-genome level. This finding suggests that these genes had a high degree of retention following WGD. More PMEs (70%) than PGs (62%) experienced segmental duplication, and more PMEs (58.2%) than PGs (43%) were retained as two or three copies in *B. rapa*. Thus, the central issue in the evolution of duplicate genes is why BraPMEs were retained more than BraPGs. One possible explanation is the functional requirement that both play important roles in plants’ developmental processes and defenses, particularly in pectin degradation, but PMEs are also involved in pectin remodeling systems^35^,^36^. This finding is consistent with the gene dosage hypothesis. In addition, we found that PME genes diverged 3.3 MYA earlier than PGs after the split with *A. thaliana* during the *Brassica*-specific WGT event. We inferred that there may have been a stronger selective pressure on PMEs than PGs that made them duplicate early to meet their survival needs, reflecting that the functions of PMEs were more varied and complex. However, how were duplication and fractionation affected in these two large gene families?

Most land plants have undergone polyploidization during their long evolutionary histories^37^,^38^. Polyploidy led to WGD and provided opportunities for duplicated genes to diverge in several evolutionary ways. Each of these genes subsequently followed one of the following three broad fates: subfunctionalization, neofunctionalization, or nonfunctionalization (deletion or pseudogenization)^39^. Some duplicate genes could also have completely redundant functions^40^. However, two different expansion types were found in the evolutionary histories of PG and PME genes.

On the one hand, previous reports have demonstrated that the cleavage of the pro domain from the mature PME might have occurred before PME functioning because it has been observed that cell-wall-extracted PMEs do not have a pro region^9^,^20^,^21^. However, the absence of a pro domain in type II PMEs did not preclude cell wall targeting^41^. Through the analyses of (i) phylogenetic relationships, (ii) gene structures, (iii) synteny analysis, (iv) nucleotide distance, and (v) ortholog groups, we found that type II B PMEs had a close relationship with type I PMEs (proPMEs), and we constructed the evolutionary model of PMEs (Fig. 5). Based on their evolutionary history, we estimated the cleavage pro domain mechanisms, which the pro domain may be modified at the genome level. In addition, both the duplicated genes in the neofunctionalization or subfunctionalization models and the expansions of the large gene family were associated with the processes of tissue-expression divergence^42^,^43^,^44^. In this study, the tissue-specific expression patterns of PME genes were also examined: most PMEs were highly expressed in flowers, particularly in pollen (*A. thaliana*). However, some PMEs of different types had similar expression patterns, indicating their common importance in plant development. The genes expressed in specific tissues might acquire new functions related to plant development, particularly flower organs. This mechanism of gene preservation should be more common in species with large effective population sizes. Due to differences in relaxed selection, the expression patterns in the group II PMEs were more divergent among the within-group than the group I PMEs. E.g., among the type II A duplicated gene pairs, some genes were found with distinct expression profiles. Based on the gene-expression patterns, we infer that the proPME duplication conforms to another form of the neofunctionalization mode. The divergent expression patterns in PMEs may contribute to the retention of these duplicated genes.

On the other hand, a previous report demonstrated that class A PGs exhibited rapid expansion in angiosperms compared with class B and C PGs^24^; however, the report did not indicate which part of the class A PGs had diverged from bryophytes and lycophytes. In this study, by examining the phylogenetic relationships among the chlorophyte, bryophyte, lycophyte and angiosperm PGs and their ortholog groups, we found that a branch of class A PGs appeared after the divergence of lycophytes and angiosperms. We divided class A into classes A1 and A2, with class A1 unique in angiosperms. Genes that are involved in hydrolase activity and that respond to external stimuli appear to have diverged rapidly after duplication, whereas those involved in nucleic acid and protein metabolism appear to have diverged slowly after duplication^45^. Notably, markedly divergent gene structures and expression patterns were also found between classes A1 and A2. New organ systems and structures in angiosperms might require more PGs to maintain their biological functions, such as in flowers and fruit. In particular, 11 *B. rapa* class A1 PGs were specifically expressed in flowers. However, most *B. rapa* class A1 PGs do not present any evidence of expression; these genes could have become pseudogenes or redundant genes after the WGD. The same is true for *A. thaliana* and *P. trichocarpa*^17^,^24^. Based on the DDC (duplication/degeneration/complementation) model^46^, these genes may be present in tissues that are not sampled or used to back up important functions in the event of a severe mutation, similar to the role of a spare tire in a car^47^. In addition, highly variable gene structures in class A1 PGs may enable them to adapt to complex environmental changes. The multiple evolutionary fates that occurred among the PGs may have contributed to the retention of these duplicate genes.

In summary, it seems reasonable to suggest that repeated WGD events facilitated the increase in PG-PME network complexity, such as in *A. thaliana* and *B. rapa* (Figure S13). By conducting a comparative evolutionary analysis with the currently available genome information in the selected plants (Figure S14), our study provides new insight into the evolutionary window of the PME and PG gene family in plants: the pro domain may have been obtained or lost in the proPMEs, and a new branch of PGs appeared after the split of gymnosperms and angiosperms during the long evolutionary history in response to developmental and environmental cues. Due to visible tissue-specific expression patterns, the expansion of PG and PME genes seems to be correlated with the evolution of increasingly complex organs in plants. This finding will lead to novel insight into functional divergence and conservation in the gene family. Further insight related to the functional differentiation in class A1 PGs and type I pro PMEs could help elucidate the significance of PG and PME evolution in plants.

## Methods

### Identification of PG and PME genes in comparison species

The *A. thaliana* PG and PME proteins were retrieved from the TAIR database (The *Arabidopsis* Information Resource, http://www.arabidopsis.org/). All files related to the *B. rapa* genome sequence data were downloaded from the *Brassica* database (BRAD; http://brassicadb.org/brad/)^25^. The gene information on *V. vinifera, C. papaya, P. trichocarpa, P. patens* and *S. moellendorffii* were downloaded from Phytozome v9.1 (http://www.phytozome.net/)^48^. The gene information on *A. trichopoda* genes were retrieved from the *Amborella* Genome Database (http://www.amborella.org/)^49^.

The Hidden Markov Model (HMM) profiles of Glycosyl hydrolase family 28 (GH28, PF00295), pectin methylesterases (PME, PF01095) and pectin methylesterase inhibitor (PMEI, PF04043) were retained from the Pfam database (http://pfam.xfam.org/). The HMM software package (hmmsearch) was used to identify the putative PG and PME proteins with the best domain e-value cutoff of 1e^−4^. To validate the HMM search, these potential sequences were analyzed using the tool SMART (http://smart.embl-heidelberg.de/)^50^ and the NCBI database (http://www.ncbi.nlm.nih.gov/). Then, to rectify incorrect start codon predictions, splicing errors, and missed or extra exons, manual reannotation was performed using the FGENESH program (http://linux1.softberry.com/berry.phtml?topic=fgenesh&group=programs&subgroup=gfind).

### Synteny analysis of PG and PME genes between *A. thaliana* and *B. rapa*

The synteny within and between *A. thaliana* and *B. rapa* was constructed using McScanX (http://chibba.pgml.uga.edu/mcscan2/; MATCH_SCORE: 50, MATCH_SIZE: 5, GAP_SCORE: –3, E_VALUE: 1E–05)^51^. An all-against-all BLASTP comparison provided the pairwise gene information and *P* values for primary clustering. Then, paired segments were extended by identifying clustered genes using dynamic programming. The positions of *B. rapa* PG and PME genes on the blocks were verified by searching for homologous genes between *A. thaliana* and three sub-genomes of *B. rapa* (LF, MF1, and MF2) at BRAD (http://brassicadb.org/brad/searchSynteny.php)^30^. The syntenic diagram was drawn using Circos software^52^. Potential duplicate genes were identified using the duplicate_gene_classifier program, which incorporates the MCScanX algorithm. The resulting blast hits were incorporated along with the chromosome coordinates of all protein-coding genes as input for this program and were classified into segmental, tandem, proximal and dispersed duplications under the default criterion. The conservation of chromosomal synteny around the PME genes in *A. thaliana* and *B. rapa* were derived from CoGe (http://www.genomevolution.org/CoGe/GEvo.pl).

### *K*_*s*_ analysis

The protein sequences of PG and PME from *B. rapa* were aligned with their syntenic genes in *A. thaliana*, which were obtained by MCScanX, using MUSCLE^53^. The protein alignments were back-translated into coding-sequence alignments using an in-house Perl script based on ParaAT^54^. *K*_*s*_ values were calculated based on the coding-sequence alignments using the method of Nei and Gojobori, as implemented in the KaKs_calculator^55^. The Ks values of all syntenic orthologs between *B. rapa* and *A. thaliana* were then plotted as histograms. The divergence time was calculated with the formula T = Ks/2R, with *K*_*s*_ being synonymous substitutions per site and R being the rate of divergence for nuclear genes from plants. R was taken to be 1.5 × 10^−8^ synonymous substitutions per site per year for dicotyledonous plants^56^.

### Phylogenetic and molecular evolution analysis of PG and PME gene family

For the phylogenetic analysis, the full-length protein sequences of PG and PME genes were aligned using the MUSCLE program with default parameters^53^. The phylogenetic tree was then constructed by the ML method using the MEGA 5.2 program^57^. The bootstrap values were calculated with 1000 replications. To estimate the nucleotide divergence between sequences, all nucleotide sequences of the PG and PME genes were also analyzed with MEGA 5.2 using the Jukes-Cantor model. Bootstrap (1,000 replicates) analyses were also performed for this estimation.

The variation in selective pressures between the different PG and PME classes were evaluated according Yang *et al*.^24^. The branch models of CODEML in PAML were used to estimate ω = (*d*_*N*_/*d*_*S*_) under two assumptions: a one-ratio model that assumes the same ω ratio for two classes and a two-ratio model in which the two classes are assigned to different ω ratios. To verify which of the models best fit the data, likelihood ratio tests (LRTs) were performed by comparing twice the difference in log-likelihood values between pairs of the models using a χ2 distribution^58^.

### Motif identification and the exon-intron structural analysis

To identify the conserved motifs of the PG and PME genes of *B. rapa*, the online Multiple Expectation-Maximization for Motif Elicitation (MEME) program, version 4.9.0^32^, was employed to identify and analyze the conserved motifs among amino acid sequences with default parameters, except for the following parameters: a maximum number of motifs set to 10 for BraPGs and 15 for BraPMEs; optimum motif width set to ≥10 and ≤100.

The gene-structure information of the PG and PME families was parsed from the General Feature Format (GFF) files of the *B. rapa* genome using an in-house Perl script. The position information of the PG, pro and PME domains was obtained from the Pfam database. Finally, the exon-intron structures and domain positions were drawn using the online program GSDS (http://gsds.cbi.pku.edu.cn/).

### Expression pattern analysis for PG and PME genes

For the expression profiling of the PG and PME genes in *B. rapa*, we utilized the Illumina RNA-seq data that were previously generated and analyzed by Tong *et al*.^59^. Six tissues of *B. rapa* accession Chiifu-401-42, including callus, root, stem, leaf, flower, and silique, were analyzed. The transcript abundance is expressed as fragments per kilobase of exon model per million mapped reads (FPKM). The gene expression patterns of each tissue were analyzed using Cluster 3.0, and the expression values were log_2_ transformed. Finally, heat maps of hierarchical clustering were visualized using Tree View (http://jtreeview.sourceforge.net/). The *A. thaliana* development expression profiling was analyzed using the AtGenExpress Visualization Tool (AVT; http://jsp.weigelworld.org/expviz/expviz.jsp) with mean-normalized values^60^. Venn diagrams were drawn using the R program.

### Orthologous PG and PME gene analysis among eight plant species

The OrthoMCL program (http://www.orthomcl.org/cgi-bin/OrthoMclWeb.cgi)^33^ was used to identify the homologous genes of PG and PME genes among the eight plant species with the default settings, which initially required an all-vs-all BLASTP. Then, the mcl clustering algorithm was used to deduce the relationship between genes. The orthologous genes were defined as genes in a cluster from at least four genes. The network of PME relationships was built using Cytoscape software^61^, and Venn diagrams of the PG ortholog groups were drawn using the R program.

## Additional Information

**How to cite this article**: Duan, W. *et al*. Comprehensive analysis of the polygalacturonase and pectin methylesterase genes in *Brassica rapa* shed light on their different evolutionary patterns. *Sci. Rep.* **6**, 25107; doi: 10.1038/srep25107 (2016).

## Supplementary Material

Supplementary Figures

Supplementary Tables
