# Supplementary material for: Comprehensive analysis of the polygalacturonase and pectin methylesterase genes in Brassica rapa shed light on their different evolutionary patterns
Source: Sci Rep. 2016 Apr 26;6:25107. doi: 10.1038/srep25107 (PMC4844994; doi:10.1038/srep25107)
Supplement: Supplementary Figures [file srep25107-s1.pdf]

**Comprehensive analysis of the polygalacturonase and pectin methylesterase genes in *Brassica rapa* shed light on their different evolutionary patterns**

**Weike Duan[1], Zhinan Huang[1], Xiaoming Song[1, 2], Tongkun Liu[1], Hailong Liu[1], Xilin Hou[1], Ying Li[1, *]**

[1]State Key Laboratory of Crop Genetics and Germplasm Enhancement/Key Laboratory of Biology and Germplasm Enhancement of Horticultural Crops in East China, Ministry of Agriculture, Nanjing Agricultural University, Nanjing 210095, China.

[2]Center of Genomics and Computational Biology, College of Life Sciences, North China University of Science and Technology, Tangshan, Hebei 063000, China.

*Corresponding author: Email: yingli@njau.edu.cn

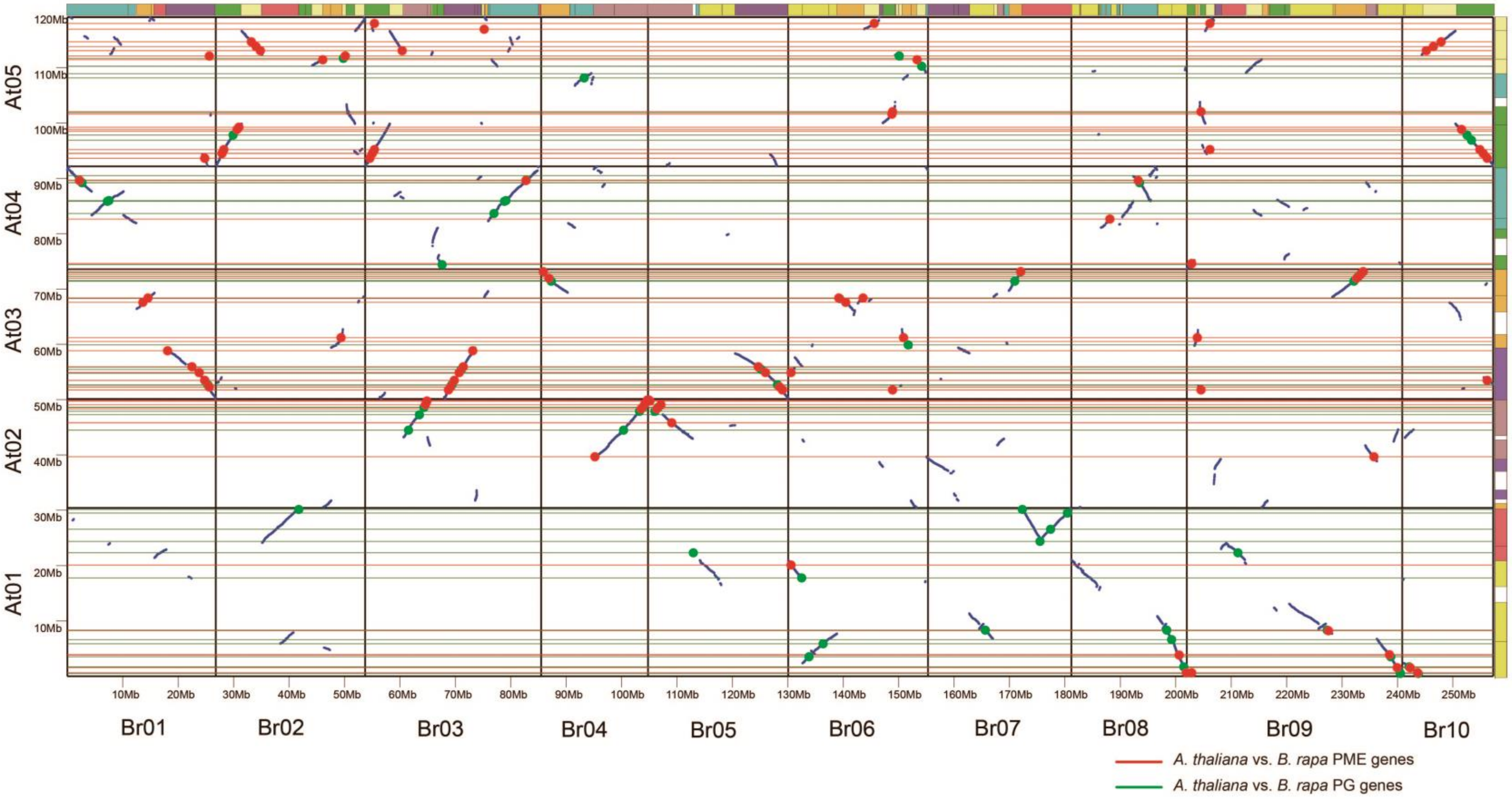


**Figure S1. Polygalacturonase (PG) and Pectin methylesterase (PME) homologous genes in segmental syntenic regions of the genomes of *Brassica rapa* and *Arabidopsis thaliana*.** Conserved collinear blocks of genes (blue irregular lines) are shown between the 10 *B. rapa* chromosomes (horizontal axis) and the five *A. thaliana* chromosomes (vertical axis). Red and green dots indicate PG and PME homologs in the two species.

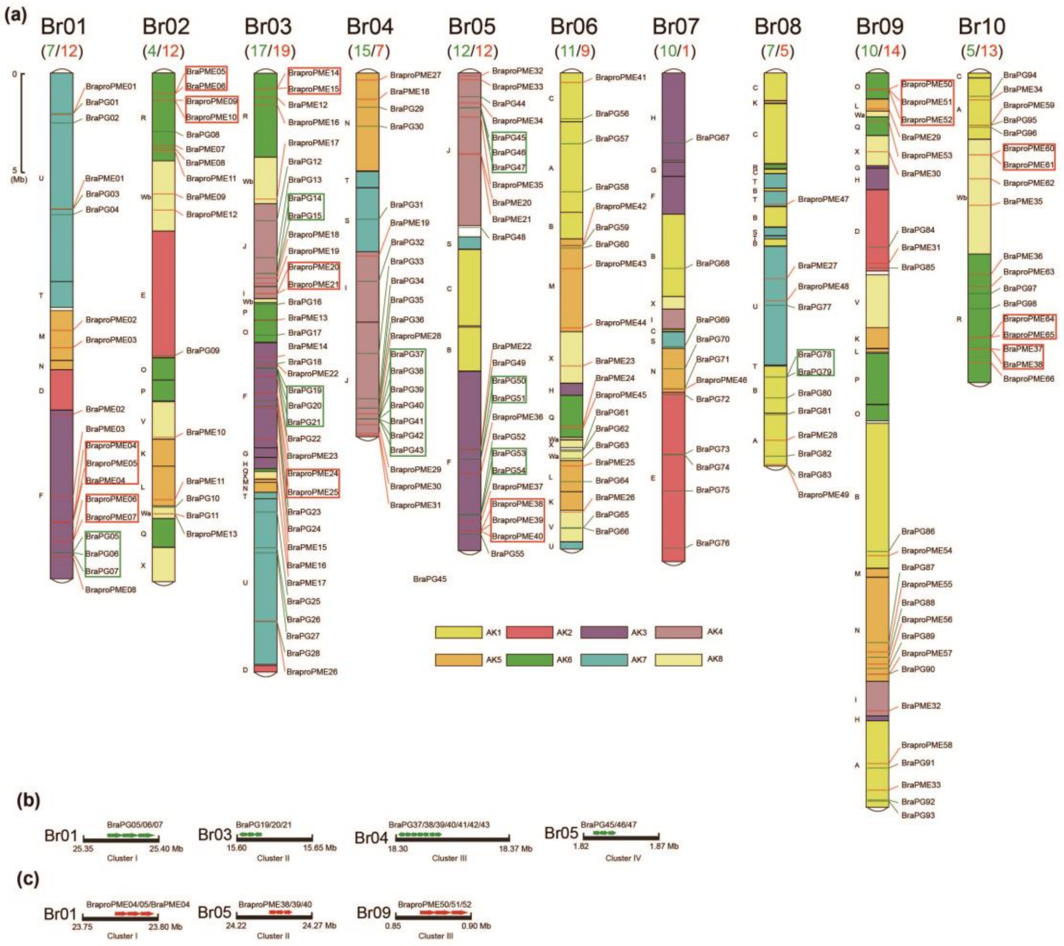


**Figure S2. Distribution of polygalacturonase (PG) and pectin methylesterase (PME) genes in *Brassica rapa* on ten chromosomes.** (a) The conserved collinear blocks on each chromosome are labeled A to X, and are color-coded according to inferred ancestral chromosomes following an established convention. Tandem duplication genes were in the boxes, while genes numbers were in the above of chromosomes. PGs and PMEs were colored green and red, respectively. The numbers of tandem duplication PG (b) and PME (c) genes were more than three in the *B. rapa* genome is shown (bottom).

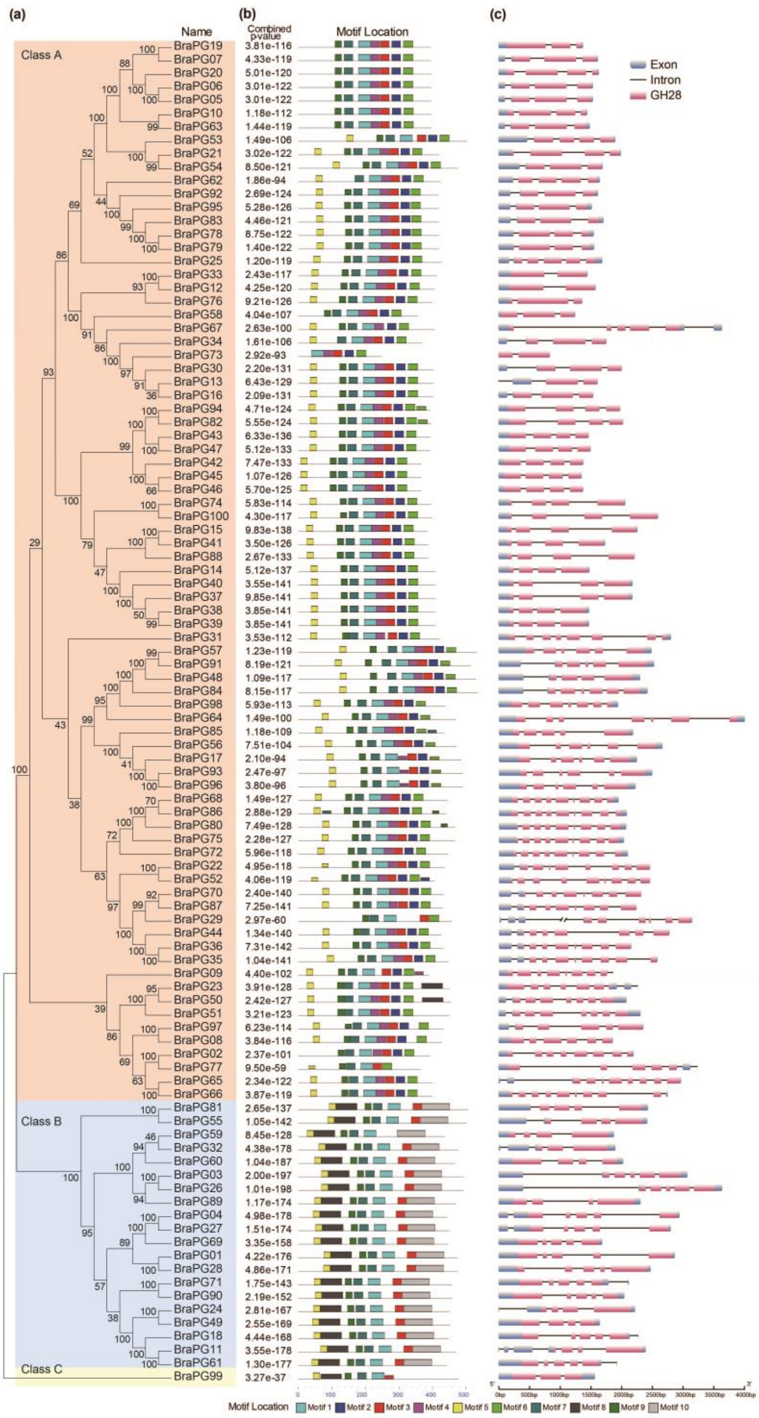


**Figure S3. An analytical view of the polygalacturonase (PG) gene family in *Brassica rapa*.** The following parts are shown from left to right. (a) Protein maximum-likelihood (ML) tree: The tree was constructed by ML method and bootstrap values were calculated with 1000 replications using MEGA5.2. (b) Protein structure: The search for the common motifs shared among the PG proteins of each group was done with MEME. Clade Situation was in the final. (C) Gene structure: the GH28 domains are highlighted by red boxes. Introns are shown as lines.

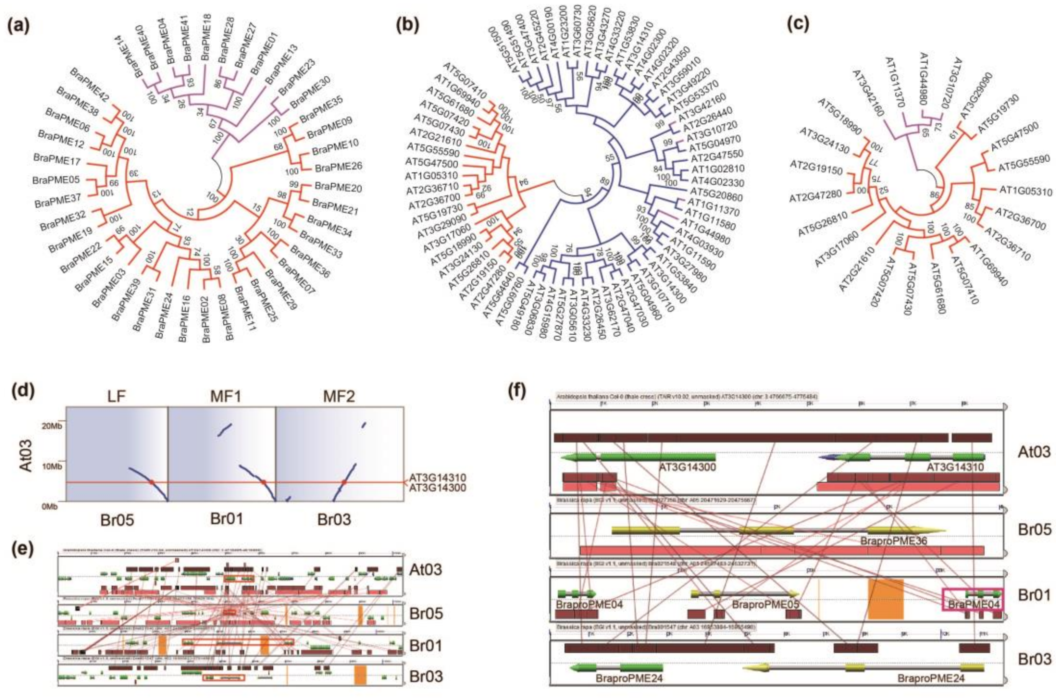


**Figure S4. Phylogenetic and syntenic relationships of pectin methylesterase (PME) genes in the *Arabidopsis thaliana* and *Brassica rapa* genomes.** (a) Protein maximum-likelihood (ML) tree of type II PMEs in *B. rapa*. (b) Protein ML tree of type I and II PMEs in *A. thaliana*. (c) Protein ML tree of type II PMEs in *A. thaliana*. In the trees, the branch of type I PMEs are colored blue, type II A PMEs are colored red and type II B PMEs are colored purple. (d) Syntenic relationships of selected PMEs in *A. thaliana* and *B. rapa* genomes. (e) (f) CoGe results of PMEs in *A. thaliana* and *B. rapa* genomes.

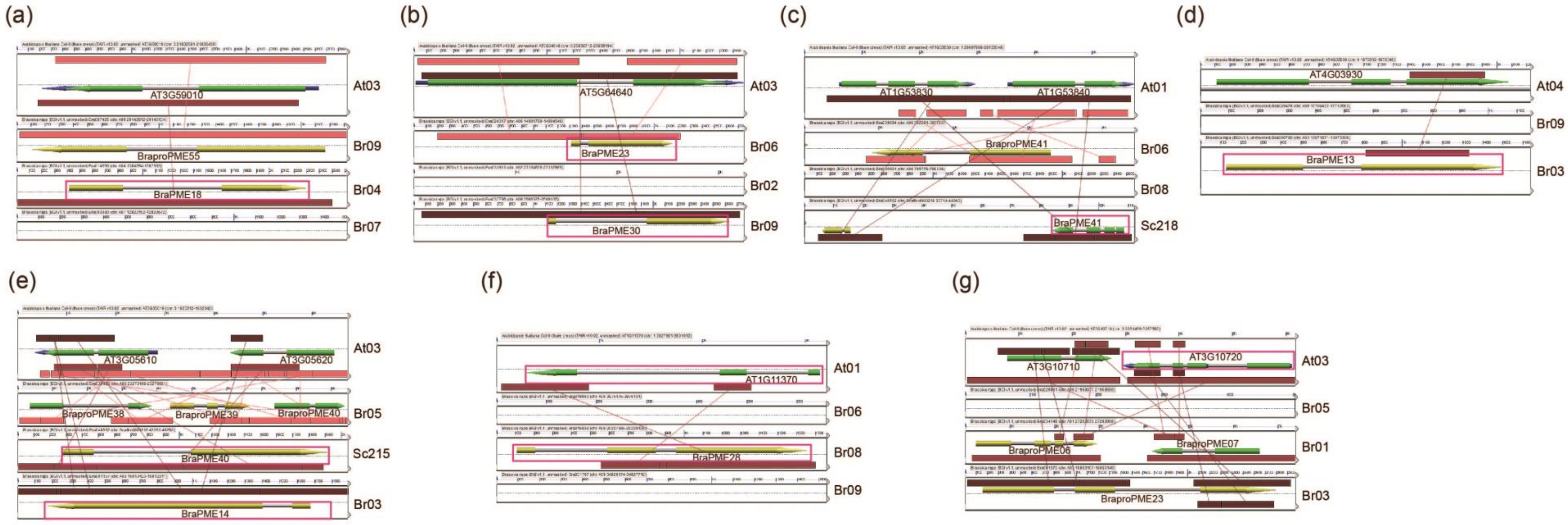


**Figure S5. Syntenic relationships of pectin methylesterase (PME) genes in the *Arabidopsis thaliana* and *Brassica rapa* genomes.** (a-g) CoGe results of PMEs in *A. thaliana* and *B. rapa* genomes: (a-e) Type II B *BraPME* genes were orthologous to type I *AtPME* genes; (f) Type II B *BraPME* gene was orthologous to the type II B *AtPME* gene; (g) Three type I *BraPME* genes were orthologous to the type II B *AtPME* gene. All the type II B PMEs are in the purple boxes.

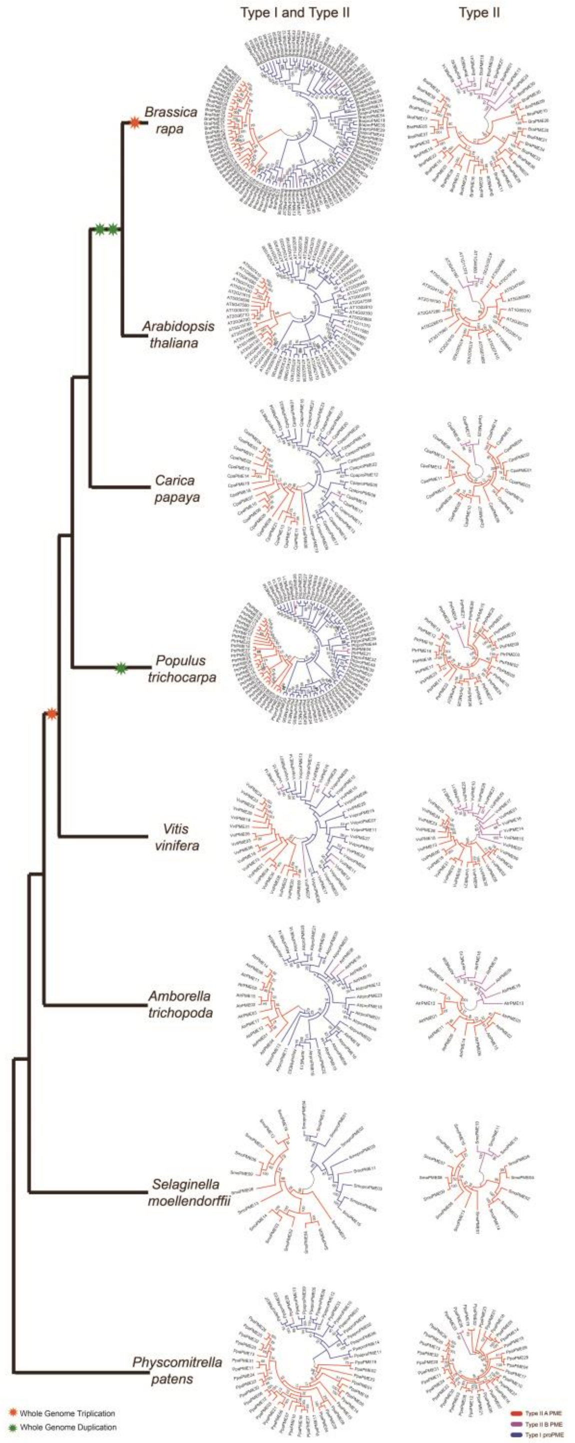


**Figure S6. Phylogenetic relationships of pectin methylesterase (PME) genes in the *Arabidopsis thaliana*, *Brassica rapa*, *Carica papaya*, *Populus trichocarpa*, *Vitis vinifera*, *Amborella trichopoda*, *Selaginella moellendorffii*, and *Physcomitrella patens*.** The α, β, γ, and salicoid duplications and the *Brassica*-specific triplication are indicated on the branches of the trees according to the Plant Genome Duplication Database. The all PMEs and type II PMEs trees were reconstructed in each species.

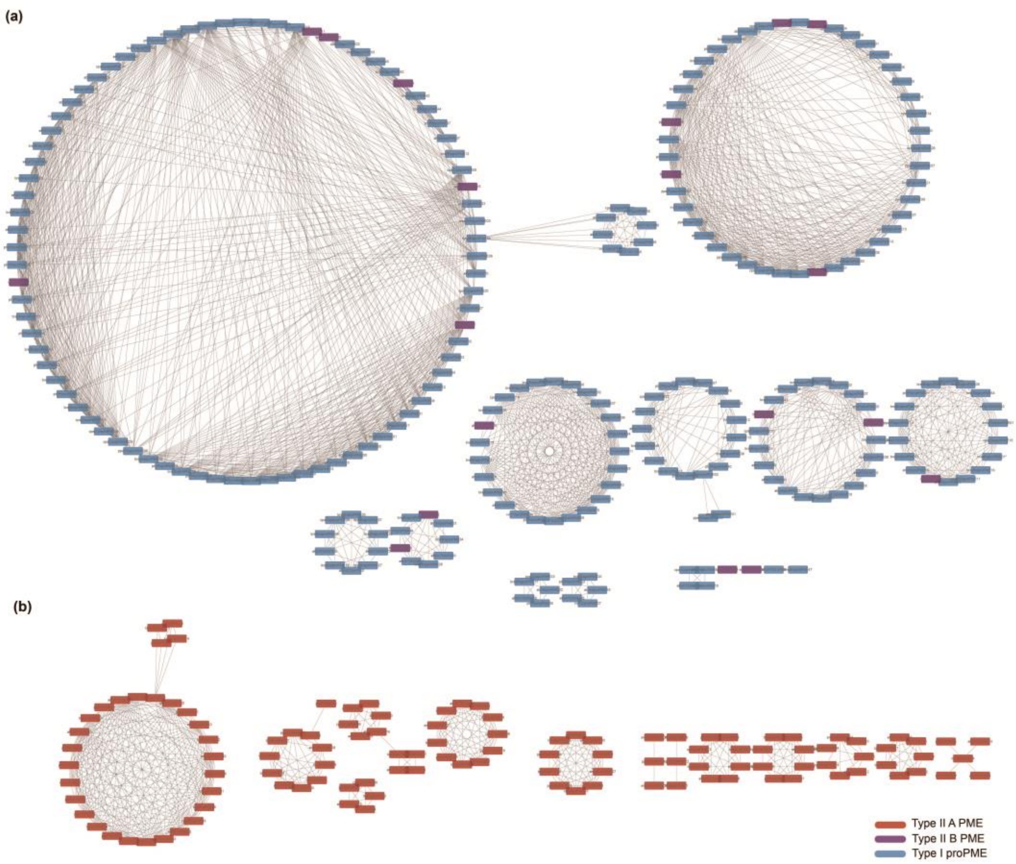


**Figure S7. The networks of pectin methylesterase (PME) genes in eight representative plant species.** This interrelation network has been constructed using these species PME orthologous gene pairs. (a) Group I: type I and type II B PMEs orthologous gene pairs networks. (b) Group II type II A PMEs orthologous gene pairs networks. The type I, type II A and type II B PMEs are highlighted by blue, red and purple boxes, respectively.

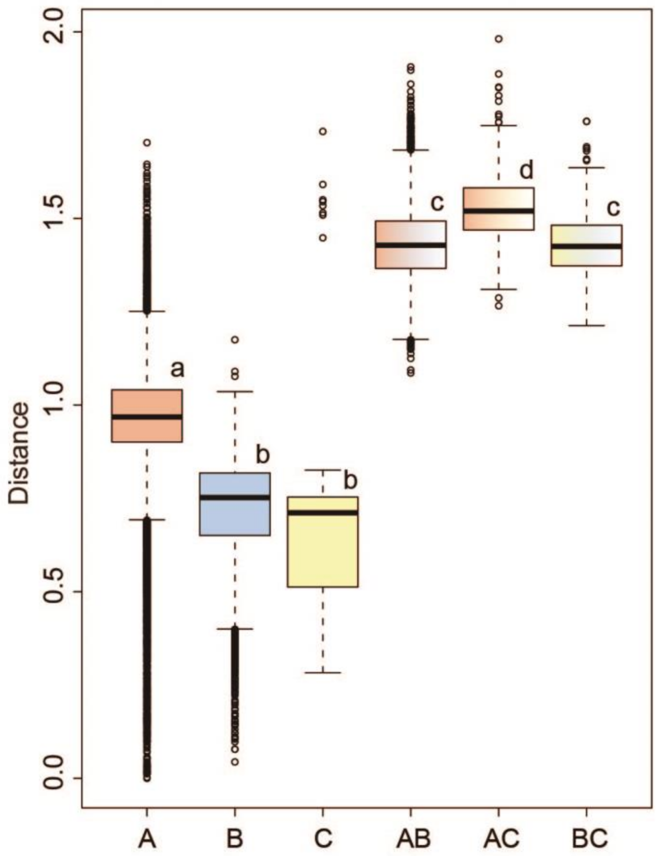


**Figure S8. Box plot of nucleotide distance among different classes of polygalacturonase (PG) genes.** The box plot shows the median (black line), interquartile range (box), and maximum and minimum scores (whiskers) of each data set. The class A, B and C PGs are highlighted by red, blue and yellow boxes, respectively. Different letters indicate statistical significance ($P < 0.05$) as determined by Duncan's Test.

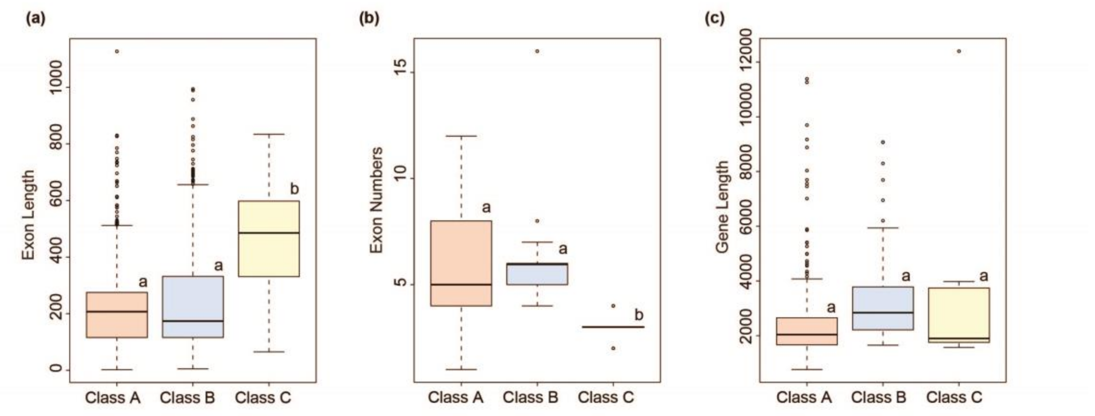


**Figure S9. Boxplot of exon length (a), exon numbers (b) and gene length (c) of the polygalacturonase (PG) genes in representative species.** The class I, II A and II B of PGs are highlighted by red, blue and yellow boxes, respectively. Different letters indicate statistical significance ($P < 0.05$) as determined by Duncan's Test.

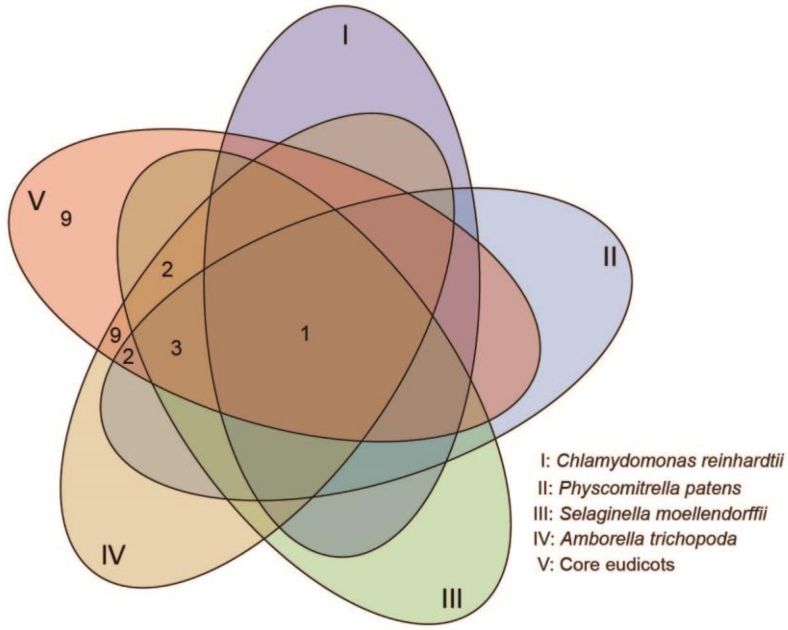


**Figure S10. The venn diagram shows the number of common and specific polygalacturonase (PG) genes ortholog groups among the follow plant species.** I *Chlamydomonas reinhardtii* II *Physcomitrella patens* III *Selaginella moellendorffii* IV *Amborella trichopoda* V Core eudicots (*Arabidopsis thaliana*, *Brassica rapa*, *Carica papaya*, *Populus trichocarpa* and *Vitis vinifera*,).

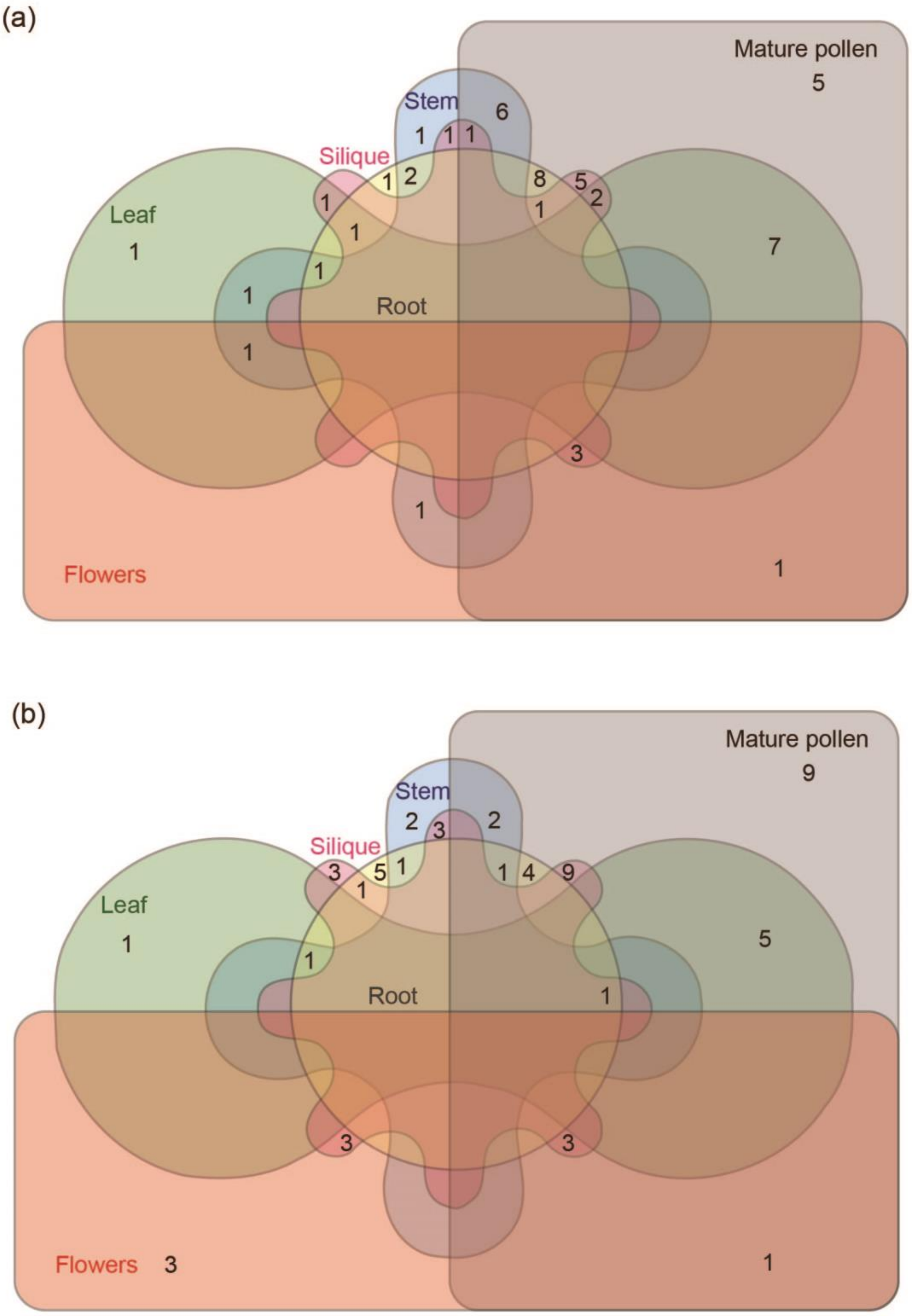


**Figure S11. Venn diagram depicting the distribution of shared expression polygalacturonase (PG; a) and pectin methylesterase (PME; b) genes among six *Arabidopsis thaliana* tissues,** i.e. root, leaf, silique, steam, flower and mature pollen

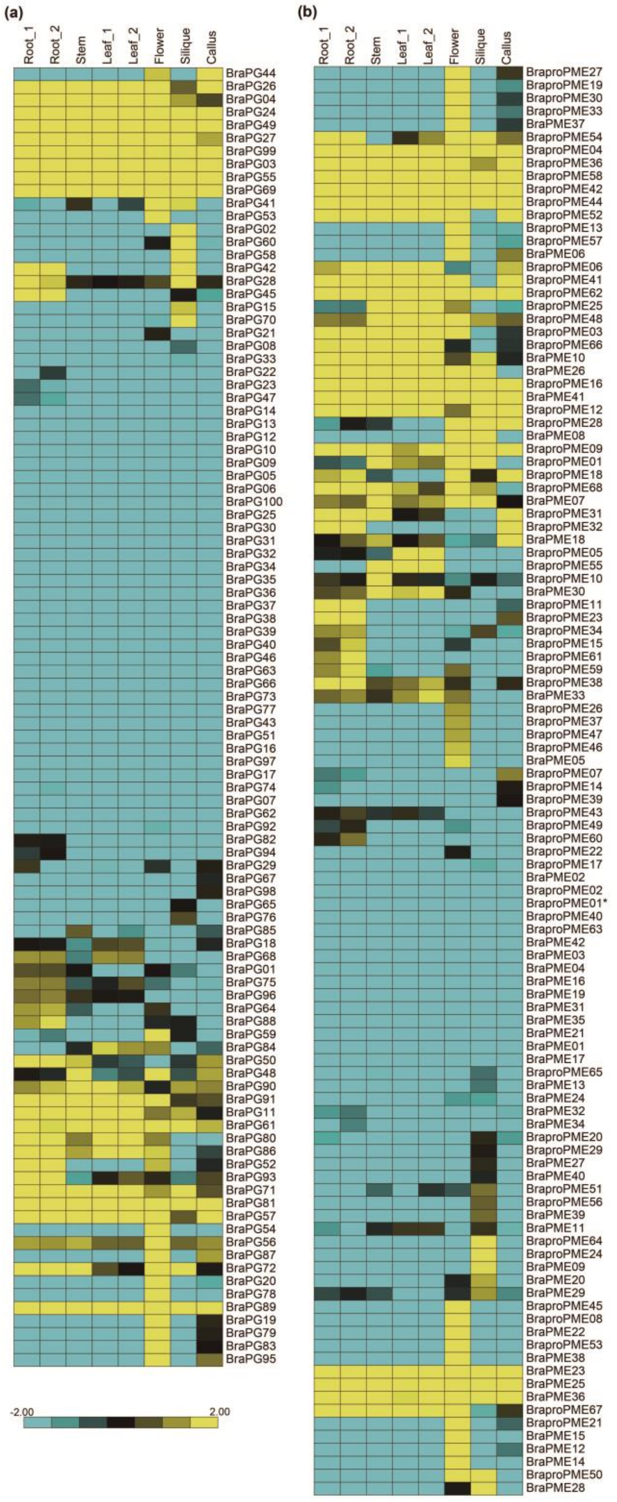


**Figure S12. Heat map representation of polygalacturonase (PG; a) and pectin methylesterase (PME; b) genes in *Brassica rapa* various tissues.** The tissues included callus, root, stem, leaf, flower, and silique. Two samples of root and leaf tissues were generated from different batches of plants. The bar at the bottom of each heat map represents relative expression values.

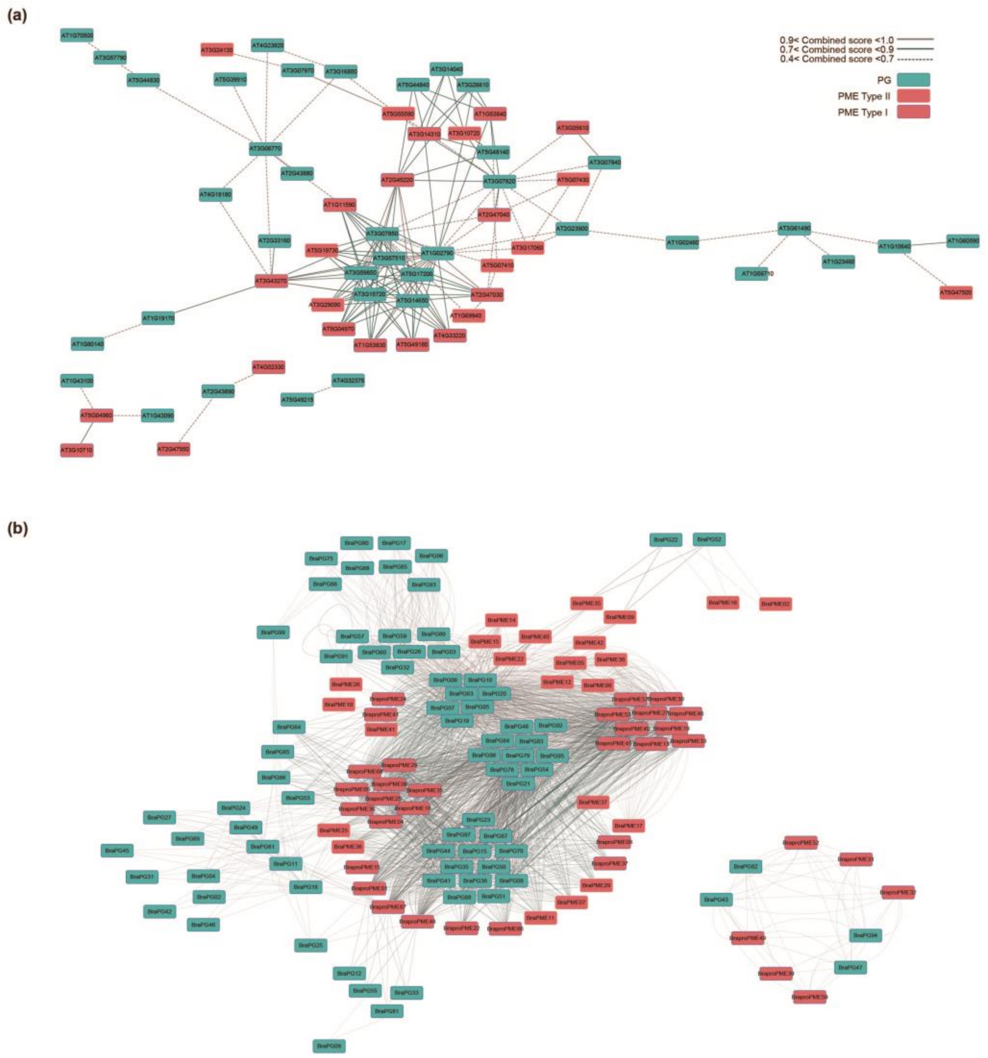


**Figure S13. Interaction network of polygalacturonase (PG) and pectin methylesterase (PME) genes in *Arabidopsis thaliana* and *Brassica rapa*.** (a) Specific protein interactions of PG-PME genes in *A. thaliana* were constructed using STRING (Search Tool for the Retrieval of Interacting Genes/Proteins; http://string-db.org/). (b) The interaction network of PG-PME genes in *B. rapa* was based on the orthologs in *A. thaliana*. The PGs and PMEs are highlighted by green and red boxes, respectively, while type I PMEs with blue edges.

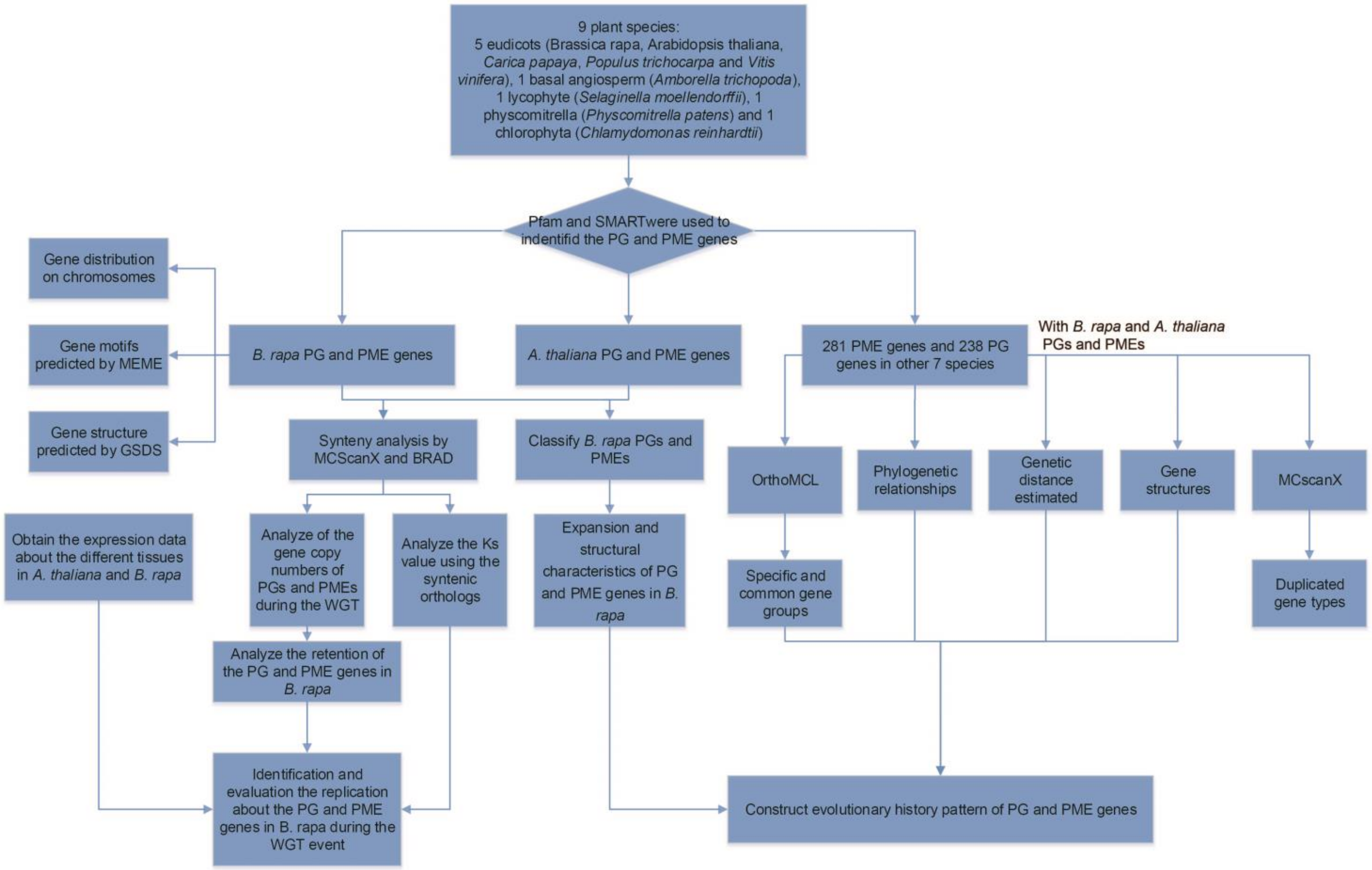


**Figure S14.** The computational pipeline of the integrated comparative genomic analysis in this study.
